# Comprehensive evaluation of total corneal refractive power by ray tracing in predicting corneal power in eyes after small incision lenticule extraction

**DOI:** 10.1371/journal.pone.0217478

**Published:** 2019-06-06

**Authors:** Chao Pan, Weina Tan, Yanjun Hua, Xiaohua Lei

**Affiliations:** 1 Hankou Aier Eye Hospital, Jianghan District, Wuhan, Hubei Province, China; 2 Department of Ophthalmology, Shanghai Jiao Tong University Affiliated Sixth People’s Hospital, Xuhui District, Shanghai, China; Nicolaus Copernicus University, POLAND

## Abstract

**Purpose:**

To assess the prediction accuracy of four variations of total corneal refractive power (TCRP) by the ray tracing method in determining corneal power in eyes after myopic small incision lenticule extraction (SMILE).

**Methods:**

Forty eyes of forty patients who had undergone myopic SMILE were enrolled in this prospective study. Manifest refraction and Pentacam HR were performed preoperatively and three months or more postoperatively. Mean keratometry (Km), true net power (TNP), equivalent keratometry readings (EKR) and 4 subtypes of TCRP (pupil centered or apex centered within a ring or a zone)—TCRP_pupil,ring_, TCRP_pupil,zone_, TCRP_apex,ring_ and TCRP_apex,zone_—were recorded and compared to the theoretical postoperative keratometry value using the clinical history method (CHM).

**Results:**

The only keratometric values that showed no statistically significant differences from the CHM were 4.0 mm and 4.5 mm EKR, 6.0 mm TCRP_pupil,zone_ and TCRP_apex,zone_. Pearson’s correlation test revealed that 4.0 mm TCRP_pupil,zone_ exhibited the highest correlation coefficient (r = 0.974) followed by TCRP_apex,zone_ 4.0 mm (0.972) and EKR 4.5 mm (0.970). The 95% limits of agreement (LOA) of the 4.0 mm EKR and CHM, the 4.5 mm EKR and CHM, the 6.0 mm TCRP_pupil,zone_ and CHM, the 6.0 mm TCRP_apex,zone_ and CHM were (-1.27 to 1.22 D), (-1.04 to 0.98 D), (-1.39 to 1.08 D) and (-1.38 to 0.96 D), respectively, while the modified 4.0 mm TCR_Ppupil,zone_ (TCRP_puil,zone_ + 0.70 D) and TCRP_apex,zone_ (TCRP_apex,zone_+0.70 D) yielded the narrowest 95% LOA of (-0.96 to 0.95 D) and (-0.96D, 1.05 D).

**Conclusions:**

Total corneal refractive power using the ray tracing method could predict corrected corneal power derived from the CHM in eyes following SMILE surgery after simple modification.

## Introduction

Currently, corneal power can be measured employing various instruments (manual and automatic keratometers, Placido-based topographers, scanning-slit technology, Scheimpflug rotating cameras, optical coherence tomographers). Considering the calculation method or optical principle involved in direct corneal power measurements, it can be categorized into three main types: the thin-lens formula, Gaussian optics formula and ray tracing method. Theoretically, the simulated keratometry (SimK) value is derived from the thin-lens formula, in which the radius of anterior corneal curvature is converted into diopter power utilizing a standardized, fictitious keratometric refractive index (usually 1.3375). Using the anterior surface to represent total corneal power without knowing posterior corneal information, it makes an essential assumption that the anterior-to-posterior ratio is 0.822 and the corneal thickness is constantly 500 μm [1]. Such an assumption works well in virginal eyes. However, in eyes after myopic corneal refractive surgery, the altered anterior surface makes the assumption invalid and corneal measurement inaccurate; thus, the keratometric value based on thin-lens formula overestimates actual corneal power measurement [2].

To overcome this dilemma, investigators have developed numerous methods, mainly in eyes following myopic photorefractive keratometry (PRK) or laser in situ keratomileusis (LASIK) [3–5]. A series of calculation formulas can increase the prediction accuracy of corneal power evaluation but can also cause confusion and waste time. In clinical practice, the keratometric values derived from the Gaussian optics formula and ray tracing method take into account the undetected radius of posterior corneal curvature in conventional keratometric reading, showing the potential to narrow the margin of miscalculation in postoperative eyes. Disappointingly, most studies investigating this subject found that total corneal power based on the Gaussian optics formula constantly underestimated actual postoperative corneal power [6–8]. In contrast, the corneal power values using the ray tracing method, for instance, total corneal refractive power (TCRP) in Pentacam (Oculus, Germany) [9, 10], mean pupil power (MPP) in Sirius (CSO, Italy)[8, 11] and total corneal power (TCP) in Galilei (Ziemer, Switzerland) [12], exhibited intrinsic advantages and potential ability in assessing postoperative corneal power accurately.

Recently, small incision lenticule extraction (SMILE) has shown promising results for correcting myopia and gained worldwide acceptance [13, 14]. The no-flap procedure largely preserves the anterior corneal surface and produces distinct changes in the anterior corneal shape compared to flap-based LASIK [15]. However, limited studies have assessed postoperative corneal power in eyes after the SMILE procedure [16]. To the best of our knowledge, no published study has assessed corneal power measured by the ray tracing method in post-SMILE eyes directly. Thus, the aim of the current study was to assess the predictability of TCRP by the ray tracing method in determining corneal power in eyes after SMILE.

## Patients and methods

### Patients

Forty eyes of forty consecutive patients with myopia and myopic astigmatism who underwent SMILE surgery at Hankou Aier Eye Hospital from September 2017 to December 2017 were prospectively enrolled into the current study. Each patient was informed of study's purpose and gave written consent to participate. The study adhered to the tenets of Declaration of Helsinki and was approved by the Hankou Aier Eye Hospital Ethics Committee. The inclusion criteria were age older than 18 years, an absence of ocular diseases other than myopia or myopic astigmatism, myopia increased no more than 0.50 D in the past 1 year, no history of hard contact lens wearing in the past 4 weeks or soft contact lens wearing in the past 2 weeks and no previous ocular trauma or surgery. The exclusion criteria were pregnancy, eyes with borderline corneal tomography or inadequate pachymetry not suitable for keratorefractive procedures, complications of corneal refractive surgery, uncorrected distance visual acuity worsen than 20/25, and follow-up less than 3 months.

### Surgical technique

SMILE procedures were performed by one experienced surgeon (XL) using a VisuMax femtosecond laser system (Carl Zeiss Meditec AG, Germany) as described in a previous study [17]. A repetition rate of 500 kHz and pulse energy of 130 Nj were utilized. The lenticule diameter (optical zone) ranged from 6.0 to 6.5 mm, and the cap diameter was 7.3 mm. The intended cap thickness was between 110 and 120 μm in all cases. At the end of the procedure, all subjects received one drop of tobramycin dexamethasone (Alcon, USA).

### Examinations

Preoperatively, all patients received a comprehensive ophthalmic examination, including uncorrected (UDVA) and corrected distance visual acuities (CDVA), manifest refraction, noncontact tonometry, slit-lamp evaluation, mydriatic fundoscopy, corneal pachymetry and Pentacam HR rotating Scheimpflug camera evaluation (version 1.20r112). Patients who met the criteria of the SMILE procedure received topical 0.5% levofloxacin (Cravit) four times a day for three days and topical 0.3% sodium hyaluronate (Hialid) four times a day for three days. Postoperative treatment included topical 0.5% levofloxacin (Cravit) four times a day for 1 week, topical 0.1% fluorometholone (Flucon) four times a day for a week and topical 0.3% sodium hyaluronate (Hialid) four times a day for three months. Manifest refraction and Pentacam HR were repeated 3 months or later postoperatively. When tested with the Pentacam HR, participants were instructed to keep both eyes open and look directly at the fixation target. Scans were taken in the automatic release mode. Among the different options available, the 25-picture scan was selected. One eye of each patient was randomly selected for the study using Microsoft Excel software to generate “1” or “2” randomly, in which “1” represented the right eye and “2” represented the left eye. Given the high repeatability for Pentacam HR in measuring corneal power parameters [18], only the first measurements with a quality specification of “OK” were used for analysis.

### Assessment of corneal power measurements with Pentacam HR

Pentacam HR Scheimpflug tomography imaging provides the following keratometric parameters:

1. Mean keratometry (Km). This value is the arithmetic mean of the pair of meridians 90 degrees apart with the greatest difference in axial power within the central 3.0 mm converted from the average anterior axial curvature (in meters) using the standard keratometric index (n = 1.3375), equivalent to SimK obtained with a keratometer or topographer. This value derives from the thin-lens formula for paraxial imagery, which considers the cornea as a fictitious single refractive surface (meant to represent both the anterior and posterior corneal surfaces) and is given by

$$Km=\left(n‐n_{0}\right)/r_{1}$$

where n is the traditional keratometric index of refraction (1.3375), n_0_ is the refractive index of air (1) and r_1_ is the radius of the anterior corneal surface (in meters).

2. True net power (TNP). This value is induced from the Gaussian optics formula for thick lenses and is calculated using the following formula according to manufacturer:

$$Corneal\;power=\left(n_{1}‐n_{0}\right)/r_{1}+\left(n_{2}‐n_{1}\right)/r_{2}$$

where n_0_ is the refractive index of air (1), n_1_ is the refractive index of the cornea (1.376), n_2_ is the refractive index of the aqueous humor (1.336), and r_1_ and r_2_ are the anterior and posterior corneal curvature (in meters), respectively. In the present study, TNP within the pupil-centered zone of 3.0 mm was recorded for analysis.

3. Equivalent keratometry readings (EKR). This value was proposed by Holladay et al. [19], intending to improve the accuracy of corneal power estimation and IOL calculation in eyes after excimer laser refractive surgery and is calculated by the following formula:

$$EKR=0.376/r_{1}‐0.03165/r_{2}$$

where r_1_ and r_2_ are the anterior and posterior corneal curvature (in meters). In Pentacam HR, EKR is displayed in the “Holladay EKR Detail Report” with a diameter of 1.0 mm, 2.0 mm, 3.0 mm, 4.0 mm, 4.5 mm, 5.0 mm, 6.0 mm and 7.0 mm. In this instance, 4.0 mm and 4.5 mm EKR were chosen for analysis.

4. Total corneal refractive power (TCRP) This value is calculated by the actual refractive index of air (1), cornea (1.376) and aqueous humor (1.336) using Snell’s law without relying on any prior assumptions. Using the ray tracing technology, the incoming parallel rays are traced through the anterior and posterior corneal surfaces, and the measured focal length is subsequently converted into the corneal power. The keratometric values are exhibited in the “Power Distribution” by the Pentacam HR with a diameter of 1.0 to 8.0 mm on a ring or over a circular area (zone) centered on the corneal apex or the pupil axis. Therefore, four variations of TCRP for a specific diameter, which include the zone centered on the pupil axis (TCRP_pupil,zone_), ring centered on the pupil axis (TCRP_pupil,ring_), zone centered on the corneal apex (TCRP_apex,zone_) and ring centered on the corneal apex (TCRP_apex,ring_), were offered and included in our analysis.

### Formulas to predict postoperative corneal power

The CHM is calculated by subtracting the refractive change at the corneal plane from the preoperative keratometry (preoperative Km used in the current study) [20].The keratometric values calculated from the following published equations were also included: (1) Haigis equivalent power formula, in which Kc = 1.119 × post Km—5.78 [4]; (2) the Shammas formula, in which Kc = 1.14 × post Km—6.8 [5]. In the present study, the CHM was adopted as the benchmark for comparisons of the various keratometric parameters obtained with Pentacam HR and the 2 proposed formulas [5–7, 9, 16].

### Statistical analysis

All the data were analyzed using MedCalc Version 11.4.2 (MedCalc Software, Belgium) and SPSS software version 25 (International Business Machines Corp., USA) for Windows. The Shapiro-Wilk test was performed to compare parameters, which are expressed as the mean ± standard deviation (SD). Comparison among all the corneal power measurements was performed using one-way analysis of variance (ANOVA) for repeated measures with Bonferroni multiple comparisons. Pearson’s correlation test was used to evaluate the relationship between series of keratometric values and the CHM value. The agreement between various corneal power measurements and the CHM was assessed by Bland-Altman plots [21]. A *P* value of less than 0.05 was considered to be statistically significant.

## Results

The mean age was 22.55 ± 4.03 years (range: 18 to 37 years). The preoperative and postoperative mean spherical equivalent (SE) were -4.91 ± 2.01 diopters (D) (range: -1.50 to -9.50 D) and 0.20 ± 0.40 D (range: -0.62 to 1.12 D), respectively. The mean preoperative Km was 42.52 ± 1.30 D (range: 39.80 to 44.50 D). The mean follow-up was 130.12±23.01 days (range: 93 to 178 days).

Table 1 displays the four variations of TCRP within a diameter of 1.0 to 8.0 mm in eyes after SMILE surgery. Significant differences among four variations of TCRP were revealed by ANOVA when diameters exceeded 5.0 mm (*P* < 0.001). Regarding the TCRP over a zone, the values presented a slight decrease from TCRP 1.0 mm to TCRP 3.0 mm as the minimum and increased to TCRP 8.0 mm gradually. No significant differences were revealed when the TCRP values were within a diameter not exceeding 7.0 mm compared to the TCRP within 1.0 mm (*P* > 0.05). Nonetheless, the difference between TCRP 7.0 mm and TCRP 1.0 mm was clinically relevant (mean difference: 0.99 D and 1.01D). TCRP measurements over a ring presented similar results. The values exhibited a slight decrease from TCRP 1.0 mm to TCRP 3.0 mm (apex centered) or TCRP 2.0 mm (pupil centered) as the minimum, then increased to TCRP 8.0 mm gradually. No significant differences were revealed when the TCRP values were within a diameter not exceeding 5.0 mm compared to the TCRP within 1.0 mm (*P* > 0.05). Nonetheless, the difference between TCRP 5.0 mm and TCRP 1.0 mm was clinically relevant (mean difference: 0.70 D and 0.77 D). Center references (corneal apex or pupil center) had no significant impact on the measurements within a specific diameter of ring or zone (*P* > 0.05).

**Table 1:** Four variations of TCRP within a diameter of 1.0 to 8.0 mm in eyes after SMILE (n = 40).

diameter	TCRP_pupil.zone_	TCRP_pupil,ring_	TCRP_apex,zone_	TCRP_apex,ring_	*F*	*P*
1.0mm	37.04±1.85	37.03±1.85	37.06±1.86	37.04±1.85	0.003	1.000
2.0mm	37.00±1.86	36.96±1.88	37.03±1.85	36.99±1.86	0.003	1.000
3.0mm	36.97±1.88	36.97±1.91	37.00±1.86	36.97±1.88	0.011	0.998
4.0mm	37.01±1.88	37.20±1.86	37.06±1.85	37.26±1.83	0.159	0.924
5.0mm	37.17±1.85	37.73±1.73	37.23±1.82	37.81±1.69	1.387	0.249
6.0mm	37.51±1.77	38.70±1.56	37.56±1.74	38.75±1.54	6.974	<0.001
7.0mm	38.03±1.66	40.08±1.50	38.07±1.64	40.10±1.49	22.488	<0.001
8.0mm	38.82±1.62	41.79± 1.64	38.77±1.56	41.85±1.61	44.622	<0.001

SMILE = small incision lenticule extraction; TCRP = total corneal refractive power

TCRP_pupil,zone_/TCRP_pupil,ring_/TCRP_apex,zone_/TCRP_apex,ring_ represent TCRP within a diameter of zone or ring centered at pupil axis or corneal apex.

**Fig 1:**
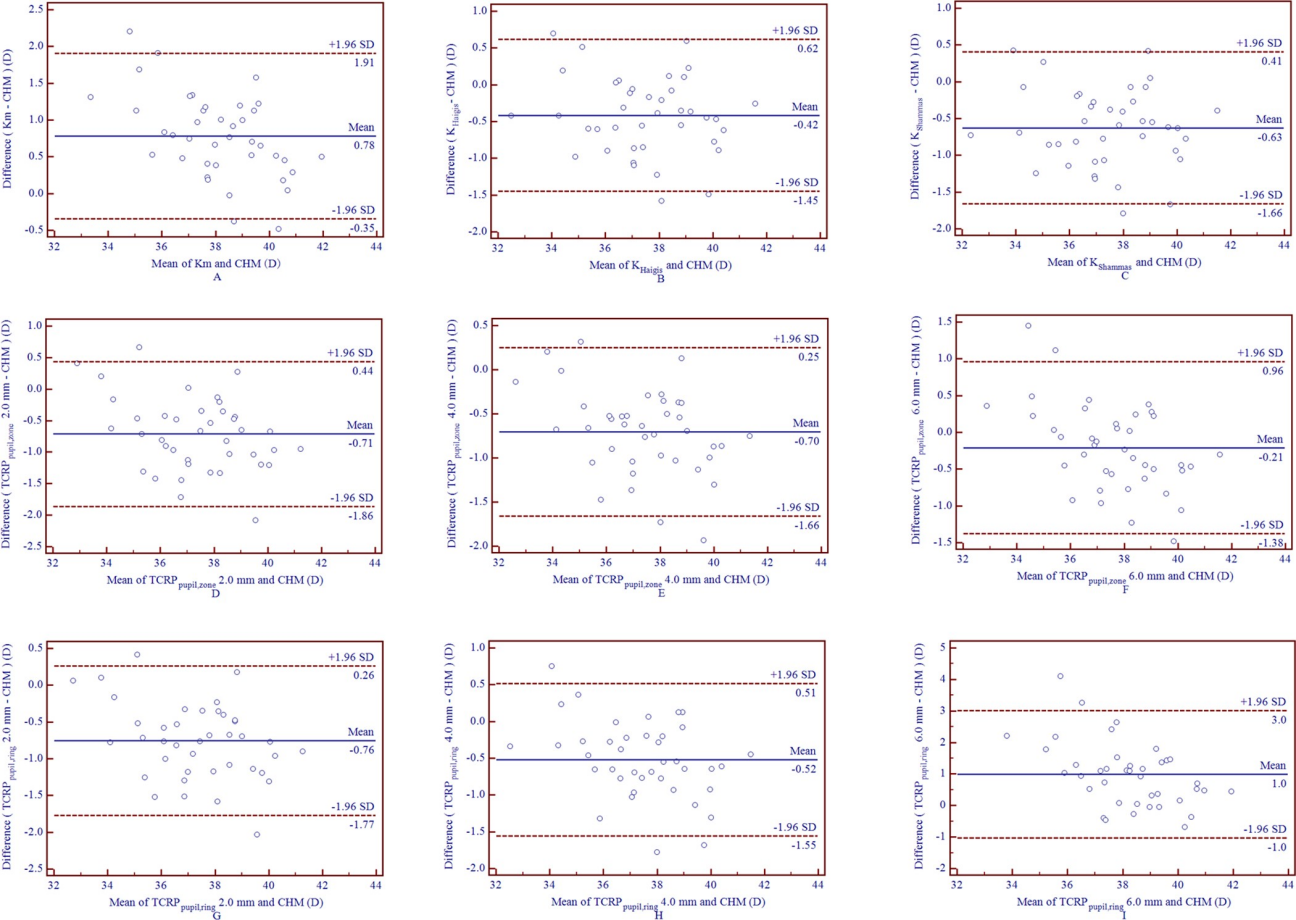
Bland-Altman plots of agreement of various corneal power measurements (Km, keratometric values derived from the 2 formulas and a series of pupil-centered TCRP) compared with the CHM value in eyes following SMILE surgery (A-I represent Km, K_Haigis_, K_Shammas_,2.0 mm, 4.0 mm and 6.0 mm TCRP_pupil,zone_, 2.0 mm, 4.0 mm and 6 mm TCRP_pupil,ring_, respectively). The solid line represents the mean difference (bias). The upper and lower lines represent the 95% LOA (the 95% LOA are shown with the dashed lines.) (CHM = clinical history method; Km = mean keratometry; K_Haigis_/K_Shammas_ represent keratometric values calculated from the Haigis method and the Shammas method; LOA = limits of agreement; SMILE = small incision lenticule extraction; TCRP = total corneal refractive power; TCRP_pupil,zone_/TCRP_pupil,ring_ represent TCRP within a diameter of a zone or ring centered on the pupil axis).

**Fig 2:**
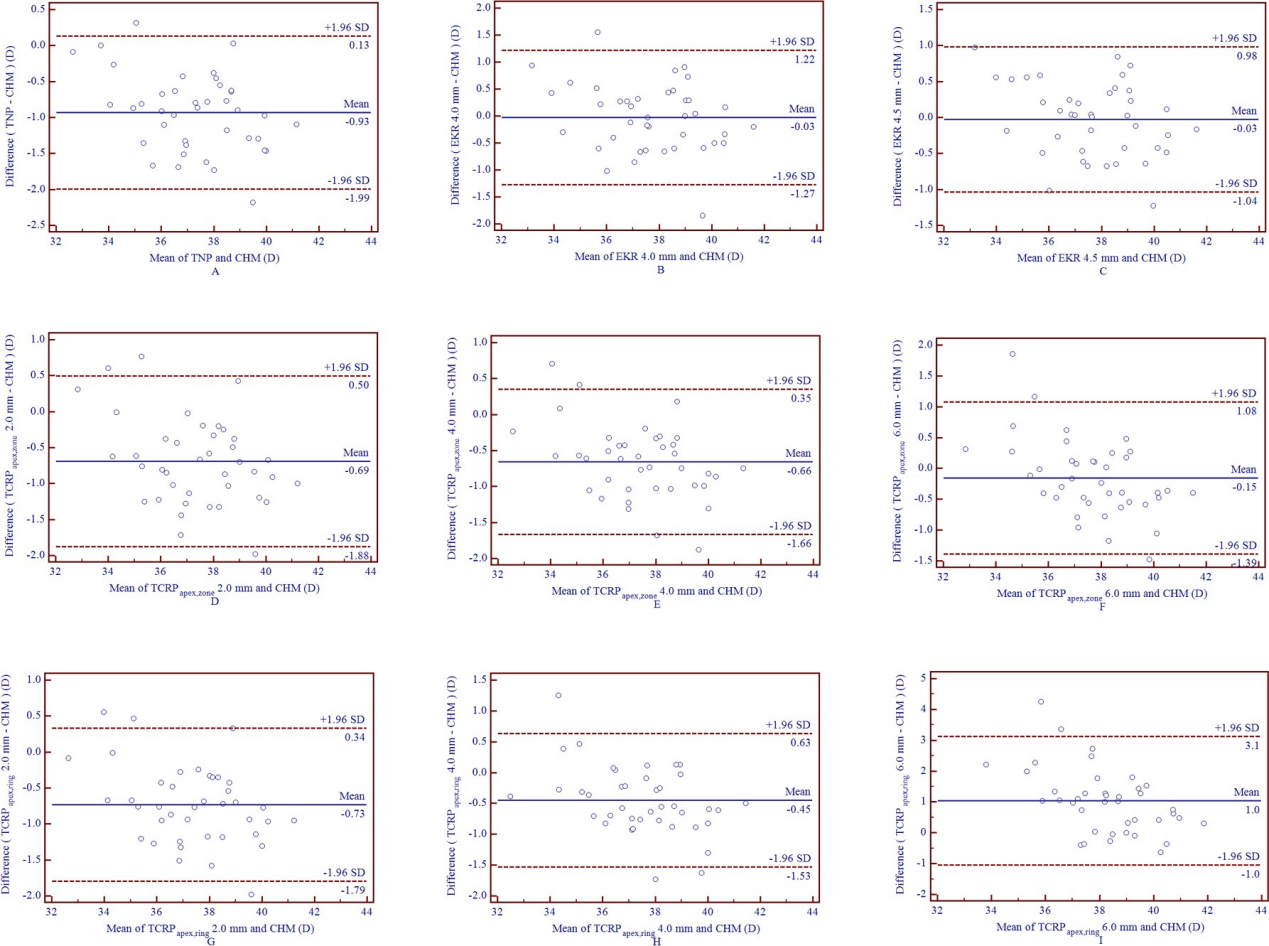
Bland-Altman plots of agreement of various corneal power measurements (TNP, EKRs and a series of apex-centered TCRP) compared with the CHM value in eyes following SMILE surgery (A-I represent TNP, EKR 4.0 mm, EKR 4.5 mm, 2.0 mm, 4.0 mm and 6.0 mm TCRP_apex,zone_, 2.0 mm, 4.0 mm and 6.0 mm TCRP_apex,ring_, respectively). The solid line represents the mean difference (bias). The upper and lower lines represent the 95% LOA (the 95% LOA are shown with the dashed lines.) (CHM = clinical history method; EKR = equivalent keratometry readings; LOA = limits of agreement; SMILE = small incision lenticule extraction; TCRP = total corneal refractive power; TCRP_apex,zone_/TCRP_apex,ring_ represent TCRP within a diameter of a zone or ring centered on the corneal apex; TNP = true net power).

Table 2 shows the comparison and correlation of four modalities of TCRP values and other keratometric parameters measured by Pentacam HR and two formulas with the CHM value. ANOVA revealed that the only keratometric parameters that had no statistically significant differences from the CHM value were the 4.0 mm and 4.5 mm EKR and the 6.0 mm TCRP_pupil,zone_ and TCRP_apex,zone_ (*P* > 0.05). Km, TCRP_pupil,ring_ 6.0 mm and TCRP_apex,ring_ 6.0 mm significantly overestimated the CHM value (*P* < 0.05), whereas TNP, the rest of the TCRP measurements and the two formula-derived keratometric values underestimated the CHM value significantly (*P* < 0.05). Pearson’s correlation test revealed high correlations between all corneal power values and the CHM, in which the TCRP_pupil,zone_ 4.0 mm showed the highest correlation coefficient (0.974),followed by TCRP_apex,zone_ 4.0 mm (0.972) and EKR 4.5 mm (0.970), whereas the TCRP_pupil,ring_ 6.0 mm and TCRP_apex,ring_ 6.0 mm presented the lowest correlation coefficients (0.868 and 0.874, respectively). Figs 1 and 2 display the agreement between various corneal power measurements and the CHM value. The 95% limits of agreement (LOA) of the 4.0 mm EKR and CHM, the 4.5 mm EKR and CHM, the 6.0 mm TCRP_pupil,zone_ and CHM, the 6.0 mm TCRP_apex,zone_ and CHM were (-1.27 to 1.22 D), (-1.04 to 0.98 D), (-1.38 to 0.96 D) and (-1.39 to 1.08 D), respectively.

**Table 2:** Comparison and correlation of four modalities of TCRP and other keratometric parameters with the CHM value in eyes following SMILE surgery (n = 40).

Corneal Power Measurement	Mean±SD (D)	Mean difference vs CHM (D)	*P* value*	95% LOA vs CHM (D)	Correlation Coefficient (r)	*P* value
CHM	37.72±2.07	—	—	—	—	—
Km	38.50±1.77	0.78±0.58	*P*<0.01	-0.35 to 1.91	0.967	*P<*0.01
TNP	36.79±1.86	-0.93±0.54	*P<*0.01	-1.99 to 0.13	0.967	*P<*0.01
EKR 4.0mm	37.69±1.90	-0.03±0.63	*P* = 1.00	-1.27 to 1.22	0.952	*P<*0.01
EKR 4.5mm	37.69±1.90	-0.03±0.51	*P* = 1.00	-1.04 to 0.98	0.970	*P<*0.01
TCRP_pupil,zone_ 2mm	37.00±1.86	-0.71±0.59	*P*<0.01	-1.86 to 0.44	0.961	*P<*0.01
TCRP_pupil,zone_ 4mm	37.01±1.88	-0.70±0.49	*P*<0.01	-1.66 to 0.25	0.974	*P<*0.01
TCRP_pupil,zone_ 6mm	37.51±1.77	-0.21±0.60	*P* = 1.00	-1.38 to 0.96	0.964	*P<*0.01
TCRP_pupil,ring_ 2mm	36.96±1.88	-0.76±0.52	*P*<0.01	-1.77 to 0.26	0.970	*P<*0.01
TCRP_pupil,ring_ 4mm	37.20±1.86	-0.52±0.53	*P<*0.01	-1.55 to 0.51	0.969	*P<*0.01
TCRP_pupil,ring_ 6mm	38.70±1.56	0.99±1.03	*P<*0.01	-1.00 to 3.00	0.874	*P<*0.01
TCRP_apex,zone_ 2mm	37.03±1.85	-0.69±0.61	*P<*0.01	-1.88 to 0.50	0.958	*P<*0.01
TCRP_apex,zone_ 4mm	37.06±1.85	-0.66±0.51	*P<*0.01	-1.66 to 0.35	0.972	*P<*0.01
TCRP_apex,zone_ 6mm	37.56±1.74	-0.15±0.63	*P* = 1.00	-1.39 to 1.08	0.960	*P<*0.01
TCRP_apex,ring_ 2mm	36.99±1.86	-0.73±0.54	*P<*0.01	-1.79 to 0.34	0.967	*P<*0.01
TCRP_apex,ring_ 4mm	37.26±1.83	-0.45±0.55	*P<*0.01	-1.53 to 0.63	0.967	*P<*0.01
TCRP_apex,ring_ 6mm	38.75±1.54	1.04±1.06	*P*<0.01	-1.00 to 3.10	0.868	*P<*0.01
K_Haigis_	37.30±1.98	-0.42±0.53	*P*<0.01	-1.45 to 0.62	0.967	*P<*0.01
K_Shammas_	37.09±2.02	-0.63±0.53	*P*<0.01	-1.66 to 0.41	0.967	*P<*0.01

CHM = clinical history method; D = diopters; EKR = equivalent keratometric readings; LOA = limits of agreement; Km = mean keratometry; K_Haigis_/K_Shammas_ represent keratometric values calculated from the Haigis method and the Shammas method; SD = standard deviation; SMILE = small incision lenticule extraction; TCRP = total corneal refractive power; TCRP_pupil,zone_/TCRP_pupil,ring_/TCRP_apex,zone_/TCRP_apex,ring_ represent TCRP within a diameter of zone or ring centered at pupil axis or corneal apex. TNP = true net power;*Bonferroni multiple comparisons with the clinical history method.

**Fig 3:**
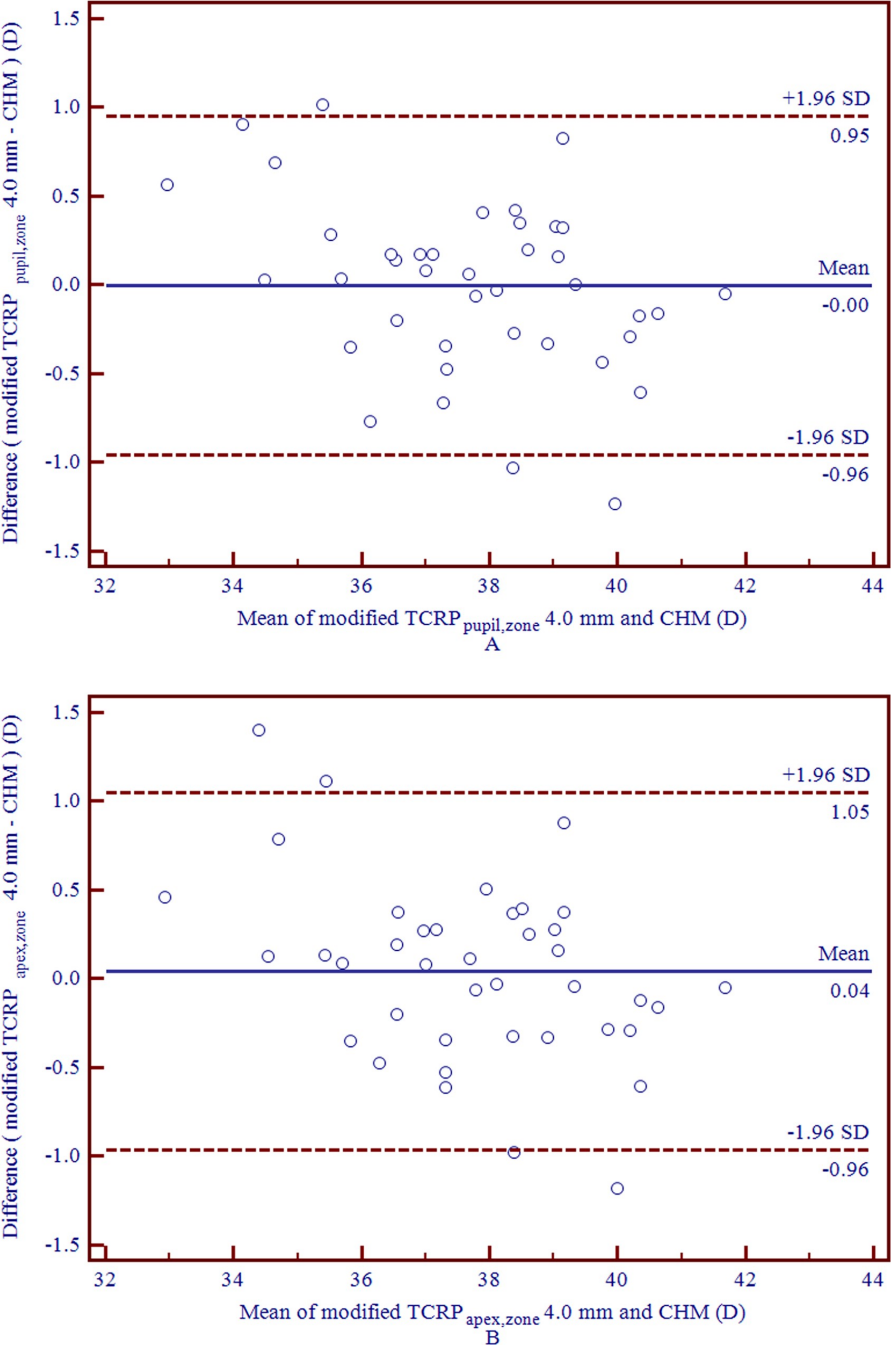
**Bland-Altman plots of agreement of modified 4.0 mm TCRP**_**pupil,zone**_ **and TCRP**_**apex,zone**_ **compared with the CHM value in eyes following SMILE surgery** (A and B represent modified 4.0 mm TCRP_pupil,zone_ and TCRP_apex,zone_, respectively). The solid line represents the mean difference (bias). The upper and lower lines represent the 95% LOA (the 95% LOA are shown with the dashed lines.) (CHM = clinical history method; LOA = limits of agreement; SMILE = small incision lenticule extraction; TCRP = total corneal refractive power; modified TCRP_pupil,zone_/TCRP_apex,zone_ represent modified TCRP within the 4.0 mm zone centered on the pupil axis or corneal apex by adding 0.70 D).

Considering that the 4.0 mm TCRP_pupil,zone_ and TCRP_apex,zone_ had the highest correlation in the current study, we converted the 4.0 mm TCRP_pupil,zone_ and TCRP_apex,zone_ into corresponding keratometric values by adding the conversion factor of 0.70 D proposed by Seo et al. [22]. The modified 4.0 mm TCRP_pupil,zone_ and TCRP_apex,zone_ had no statistically significant differences compared to the CHM values (0.00 D and 0.04 D), while the 95% LOA were (-0.96 D, 0.95 D) and (-0.96D, 1.05 D), respectively, as shown in Fig 3.

## Discussion

Corneal power measurement in eyes after myopic corneal refractive surgery has frustrated ophthalmologists for more than two decades. As a consequence, numerous studies have focused on the accurate assessment of postoperative corneal power and proposed more than 20 methods to solve this issue [2–5]. The ray tracing method shows the potential to precisely determine corrected corneal power in eyes after myopic keratorefractive surgery compared to the SimK based on the thin-lens formula and the total corneal power derived from the Gaussian optics formula, which has been verified in eyes after PRK/LASIK in series of studies [8–12]. However, few studies have paid attention to eyes following the SMILE procedure, which shows distinct anterior corneal surface changes compared to flap-based LASIK [15]. Thus, the aim of this study was to evaluate the performance of the ray tracing method in predicting corneal power after SMILE using Pentacam HR.

We first evaluated the distribution characteristics of four variations of TCRP from the central to the peripheral cornea. In contrast to previous studies[17], in which TCRP increased gradually from 1.0 mm to 8.0 mm because of the presence of spherical aberration, TCRP in our study decreased from a diameter of 1.0 mm to 3.0 mm as the minimum value (except for TCRP_pupil,ring_ with 2.0 mm as the minimum), then increased gradually to a diameter of 8.0 mm. We presume that the slight increase of spherical aberration within the central 3.0 mm may have been counteracted by the central corneal shape change induced by the surgical procedure. Further studies are required to explore the potential mechanism. The measurement-centered reference (pupil axis or corneal apex) plays an essential role in the detection and interpretation of corneal power assessments. Previous studies [9–11, 17, 22–24] mostly involved the pupil-centered instead of corneal apex-centered TCRP in their analyses based on the concerns that the entrance pupil controls the light rays propagated through the ocular refractive media and represents the most accurate corneal power measurement reference from the perspective of optical transmission. In our study, the centered reference had no significant impact on the TCRP value within a specific diameter over a ring or in a zone, which resonates with former investigations. Næser et al. [25] explored four modalities of TCRP in 951 normal eyes and found no significant difference using the pupil axis versus corneal apex centration. However, caution should be used considering that Pentacam Scheimpflug cameras may miscalculate the pupil location in normal or postoperative eyes or that a high kappa angle can be present in a specific individual. In eyes after corneal refractive surgery, decentered treatment may be occasionally encountered.

In the part of corneal power estimation, we only included four modalities of TCRP within a diameter of 2.0 mm, 4.0 mm and 6.0 mm to represent the central, paracentral and peripheral cornea, respectively. To the best of our knowledge, this report describes the first study to compare TCRP measured by the ray tracing method with the CHM value directly in eyes after the SMILE procedure. Encouragingly, 6.0 mm TCRP_apex,zone_ and TCRP_pupil,zone_ had no significant differences (-0.15 D and -0.21 D) compared to the CHM value. However, the 95% LOA (-1.39 to 1.08 D and -1.38 to 1.06 D) were wide, indicating that errors may arise when using the 6.0 mm TCRP_apex,zone_ and TCRP_pupil,zone_ to predict the theoretical postoperative corneal power. Qian et al. [17] stated that the functional optical zone is approximately 1.5 mm smaller than the size of the lenticule planned to be extracted in SMILE surgery. This finding may suggest that the functional optical zone in the current study may be around 4.5 to 5.0 mm, considering that the sizes of lenticule extracted in the SMILE procedure were 6.0 to 6.5 mm. Therefore, the 6.00 mm TCRP_apex,zone_ and TCRP_pupil,zone_ values may include the areas outside the function optical zone and introduce prediction errors, which could also be extrapolated from the result that the 6.0 mm TCRP_apex,ring_ and TCRP_pupil,ring_ exhibited the worst agreement and lowest correlation. TCRP values within smaller diameters have been adequately investigated in eyes after myopic laser refractive surgery, mainly to predict the surgically induced refractive change, and have yielded diverse results, including the 2.0 mm TCRP_pupil,ring_[10], 3.0 mm TCRP_apex,ring_[23] and TCRP_pupil,zone_ over a diameter of 2.0 to 5.0 mm [9, 10, 17]. The homogeneity of the selected sample, the principle of surgical procedures, and the version of the Pentacam software (1.17r89, 1.18r15 and others not mentioned) may contribute to the discrepancy in the findings of previous studies. Similarly, investigations focusing on the prediction accuracy of TCRP in estimating the postoperative theoretical keratometric value using CHM exhibited varying findings. Oh et al. [9] reported that TCRP_pupil,zone_ (0 mm, 1.0 mm, 3.0 mm and 4.0 mm) showed no significant differences compared with the CHM value (-0.02 D, -0.05 D, -0.17 D and -0.06 D, respectively) in eyes after PRK, in which the narrowest 95% LOA was obtained by the 4.0 mm TCRP_pupil,zone_ (-1.20 to 1.22 D). In contrast, Ng et al. [24] revealed that 4.0 mm TCRP_puil,zone_ significantly underestimated the CHM value by 0.53 D using Pentacam AXL in eyes after LASIK. Similar results were found by Seo et al. [22] (0.60 D). To address this problem, Seo et al. [22] proposed adding a conversion factor of 0.70 D to transfer TCRP into the conventional keratometric reading used directly in traditional IOL calculation and got satisfactory results. Interestingly, the TCRP within the zone of 4.0 mm diameter, either centered on the pupil axis or the corneal apex, significantly underestimated the CHM value by approximately 0.70 D in the present study (0.70 D and 0.66 D, respectively). If we obtained a modified TCRP by just adding 0.70 D, the adjusted 4.0 mm TCRP_pupil,zone_ and TCRP_apex,zone_ had no significant differences compared to the CHM value (0.00 D and 0.04 D, respectively). The 95% of LOA were (-0.96 D, 0.95 D) and (-0.96D, 1.05 D), which were better than previous studies in eyes after PRK/LASIK and were the best among the miscellaneous evaluated keratometric values in the current study. This result may represent the most important finding in our study, which confirms that TCRP using the ray tracing method has potential advantages in assessing corneal power in eyes after SMILE surgery. However, conversion must be performed before being used as the traditional keratometric value or entered into the conventional IOL calculation formulas. The conversion factor has to be verified in a large sample size to confirm the current conclusions. Recently, a large clinical material study (951 normal eyes) conducted by Næser et al. [25] reported that 4.0 mm TCRP_pupil,zone_ significantly underestimated SimK by 0.50 D, which was slightly lower than the results reported by Seo et al. [22] (0.70 D for virgin corneas and 0.60 D for postoperative corneas). A large clinical observation study in China may be warranted to further explore the potential bias and increase the assessment ability of TCRP in eyes after corneal refractive surgery.

The equivalent keratometry readings were primarily proposed by Holladay et al. [19] to narrow the margin of prediction error in total corneal power and improve the accuracy of IOL power calculation in eyes after corneal refractive surgery, and 4.5 mm EKR was recommended for clinical practice, which yielded the closest resemblance to the keratometric value obtained by CHM (-0.06±0.56 D). Similar results have been reported by Falavarjani et al. [7] with a relatively wide 95% LOA (-1.65 to 1.17 D). Conversely, several studies reported that the 4.5 mm EKR significantly overestimated postoperative corneal power in eyes after PRK or LASIK/LASEK compared to CHM (range from 0.62 D to 0.70 D) [6, 26, 27]. Recently, Ng et al. [24] reported that the 4.0 mm EKR had no significant difference from the CHM (0.087D), with moderate 95% LOA (-1.10 D, 1.28 D) in post-LASIK eyes, while the 4.5 mm EKR had a statistically significant but not clinically significant difference compared to the CHM (0.156 D), with slightly wider 95% LOA (-1.05 D, 1.36 D). Interestingly, the narrowest agreement was obtained in eyes after the SMILE procedure: Wei et al. [16] reported that the 4.0 mm and 4.5 mm EKR had 95% LOA of (-0.94 D,0.90 D) and (-0.83 D, 0.88 D), respectively, with mean differences of -0.023 D and 0.027 D in comparison to the CHM reading. Our result (-0.03 D and -0.03 D), with the 95% LOA of (-1.27 D, 1.22 D) and (-1.04 D, 0.98 D), is consistent with those findings, which further enhances the impression that the EKR has better performance in predicting total corneal power in post-SMILE eyes than that in post-LASIK eyes. Previously, Gyldenkerne et al. [15] found that the no-flap procedure could largely preserve the anterior corneal surface and produce distinct changes in the anterior corneal shape compared to flap-based LASIK. Holladay et al. [19] reported that the EKR might depend on the type of ablation. Therefore, the superior performance of EKR in post-SMILE eyes may be partly explained. The heterogeneous patient group, including a mixture of myopic PRK, LASIK and LASEK in previous studies [6, 26], may also be related to the inferior performance.

To date, only one study has evaluated the prediction accuracy of TCRP and EKR simultaneously in eyes following corneal refractive surgery. Ng et al. [24] found that the 4.0 mm EKR demonstrated closer agreement with the value derived with the CHM compared to 4.0 mm TCRP_pupil,zone_ (-1.10 to 1.28 D versus -0.88 to 1.95 D). Similar results are reported in our study. However, it cannot simply be concluded that the EKR is superior to TCRP in assessing corneal power in eyes after myopic refractive surgery, considering that EKR is an adjustment of the corneal power calculated using the Gaussian optics formula and comprising a conversion factor so that it can be directly used in traditional IOL formulas, which assume n = 1.3375 (such as Holladay and SRK/T) [19], whereas TCRP is directly derived from the ray tracing method through the anterior and posterior corneal surface using Snell’s law of refraction, which could not be used routinely in the conventional corneal power measurement and IOL power calculation without modification and validation. If we also converted 4.0 mm TCRP_pupil,zone_ and TCRP_apex,zone_ into corresponding keratometric readings by adding the correction factor of 0.70 D mentioned above, the 95% LOA (-0.96 to 0.95 D and -0.96 D to 1.05 D) were even better than the 4.0 mm and 4.5 mm EKR. It is not surprising that TCRP presents a potential advantage to predict theoretical postoperative corneal power more accurately. TCRP, which takes into account the anterior and posterior corneal curvature, the corneal thickness and the refractive effect without relying on any assumptions, represents the most accurate corneal power measurement [17]. However, traditional corneal power measurements are based on paraxial optics thin-lens formula using a fictitious keratometric index to convert the anterior corneal curvature into the equivalent corneal power. Therefore, the TCRP should be customized with some type of modification before being used routinely as an equivalent corneal power.

As a secondary outcome, the present study assessed the agreement of corneal power evaluation with two methods developed by prior investigators and the CHM. The Haigis method and the Shammas method underestimated the CHM value by 0.42 D and 0.63 D, respectively. Similar results have been reported by Wei et al. [16], in which the differences were slightly lower (0.17 D and 0.36 D). The Shammas method is a clinically derived method based on a regression formula between the CHM and postoperative keratometric values in post-LASIK eyes [5], which was expected to show the best performance in the current study. Nevertheless, the significant difference and the wide LOA (-1.66 to 0.41 D) indicated that the Shammas method is not a compelling alternative to the CHM method in eyes following SMILE surgery. The Haigis equivalent power formula is a theoretical equation derived from performing model calculations on a myopic Gullstrand eye using customized computer programs [4]. Wide agreement was exhibited between the Haigis method and the CHM, with a 95% LOA of -1.45 to 0.62 D, indicating that caution should be taken when the Haigis method is used as an alternative to the CHM.

The present study has limitations. First, we only evaluated a small sample of subjects with myopic SMILE surgery. Further studies with a larger sample of subjects are warranted to confirm the current results. Second, the benchmark chosen in our study, the CHM, might not be accurate because the preoperative data could be imprecise or unstable due to either inaccurate measurements or interval changes in the corneal curvature or lens power and clarity. Therefore, increasing evidence [3, 28] casts doubt on the accuracy of the CHM values and suggests the back-calculation of corneal power from the actual IOL outcomes should be the new gold standard. However, our study has investigated post-SMILE eyes in short-term follow-up and intended to find an alternative method to the CHM. The limitation of CHM itself has been controlled to the mildest level and might have had little impact on the accuracy of our results. SMILE surgery candidates were relatively young in our study due to the relative newness of the surgical procedure, so subsequent cataract surgery may not be needed in the near future.

In conclusion, total corneal refractive power using the ray tracing method has the potential to predict corrected corneal power derived from the CHM in eyes following SMILE surgery. Modification with a correction factor is strongly recommended to ensure precise estimation. Further studies in a larger population are warranted to explore and identify the appropriate conversion factor.

## Supporting information

S1 Checklist STROBE checklist v4 combined PlosMedicine. (DOCX)

S1 Data Original data. (XLSX)
